# Non-negligible Contributions to Thermal Conductivity From Localized Modes in Amorphous Silicon Dioxide

**DOI:** 10.1038/srep35720

**Published:** 2016-10-21

**Authors:** Wei Lv, Asegun Henry

**Affiliations:** 1 George W. Woodruff School of Mechanical Engineering, Georgia Institute of Technology, Atlanta, GA 30332, USA; 2 School of Materials Science and Engineering, Georgia Institute of Technology, Atlanta, GA, 30332, USA

## Abstract

Thermal conductivity is important for almost all applications involving heat transfer. The theory and modeling of crystalline materials is in some sense a solved problem, where one can now calculate their thermal conductivity from first principles using expressions based on the phonon gas model (PGM). However, modeling of amorphous materials still has many open questions, because the PGM itself becomes questionable when one cannot rigorously define the phonon velocities. In this report, we used our recently developed Green-Kubo modal analysis (GKMA) method to study amorphous silicon dioxide (a-SiO_2_). The predicted thermal conductivities exhibit excellent agreement with experiments and anharmonic effects are included in the thermal conductivity calculation for all the modes in a-SiO_2_ for the first time. Previously, localized modes (locons) have been thought to have a negligible contribution to thermal conductivity, due to their highly localized nature. However, in a-SiO_2_ our results indicate that locons contribute more than 10% to the total thermal conductivity from 400 K to 800 K and they are largely responsible for the increase in thermal conductivity of a-SiO_2_ above room temperature. This is an effect that cannot be explained by previous methods and therefore offers new insight into the nature of phonon transport in amorphous/glassy materials.

Understanding the underlying physics of thermal conductivity (TC) in crystalline dielectric materials has made a great progress in recent years^1^,^2^,^3^,^4^,^5^,^6^,^7^,^8^,^9^. Using expressions based on the phonon gas model (PGM)^10^,^11^ and first principles calculations, it is now possible to predict the TC of dielectric crystalline materials very accurately^4^,^12^,^13^. Furthermore, the PGM has guided our understanding of phonon transport in micro and nano-structures and has provided a more than satisfactory explanation of the behavior in a variety of systems^14^,^15^,^16^. As a result, it has rightfully become our primary lens for interpreting phonon transport related phenomena. However, disordered materials such as amorphous silica, studied herein, are much less well understood and models that reproduce experiments are lacking.

From the PGM perspective, TC is described by the phonon contributions to the specific heat, the phonon group velocities and mean free paths (MFPs)^17^,^18^. First, the specific heat can be calculated from a lattice dynamics (LD) calculation of the phonon frequencies^18^,^19^. Second, the phonon velocities can also be obtained from LD calculations of the dispersion *ω*(*k*), where the group velocity is given by d*ω*/d**k**. Lastly, the MFP, which is the product of the group velocity and time between phonon scattering events (e.g., the relaxation time), can be calculated from normal mode analysis^2^,^20^,^21^ or first principle calculations^3^,^12^,^13^,^22^. For crystalline compounds, the system is periodic, which allows for straightforward definition of the dispersion and thus the group velocity is well defined. However for amorphous materials, or other structurally/compositionally disordered systems, due to the lack of periodicity, one cannot clearly define the group velocity^23^. As a result, since the PGM hinges on knowledge of the group velocities, application of the PGM to amorphous materials requires non-rigorous assignments of group velocities and is therefore highly questionable. Several studies^21^,^24^ have related the behavior of low frequency modes in amorphous materials (e.g., propagons) to propagating modes in crystalline materials, because they exhibit plane-wave modulated vibrations. The majority of the modes in amorphous materials, however, are non-propagating modes (e.g., diffusons and locons)^25^ and as a result, MFP based explanations of phonon transport in amorphous materials become difficult to rationalize.

In light of this issue, the seminal work of Allen and Feldman (A-F)^26^, which expressed the TC of amorphous materials based on a mode diffusivity instead of MFP has therefore become widely accepted in understanding the phonon transport in amorphous materials, since it does not require one to define the phonon velocity. However, its limitation is that it does not include the effects of anharmonicity in the atomic interactions. Thus, it is interesting to consider how anharmonicity might affect the contributions of different modes in a material such as amorphous silica (a-SiO_2_), for which the A-F method does not exhibit satisfactory agreement^27^ with experiments. Here it is important to note that the only temperature dependence in the A-F model comes from the temperature dependent specific heat, while the Green-Kubo Modal Analysis (GKMA) method, employed herein is also able to include the temperature dependence of the mode diffusivities and frequencies.

Silica is also of widespread technological significance and therefore understanding its thermal transport physics is of significant and broad interest. Of particular importance is the fact that the TC of a-SiO_2_ increases with temperature beyond room temperature, which is somewhat difficult to explain with MFP based arguments. Furthermore, the model developed by A-F, for example, predicts essentially constant TC above 200 K^27^ and therefore it is important to examine the role of anharmonicity in the TC of a-SiO_2_.

Recently Lv and Henry^23^ developed a new method termed the GKMA method, which is a combination of Green-Kubo (GK) formula with modal information from LD. The GKMA method is significant because it incorporates all degrees of anharmonicity, since it uses molecular dynamics (MD) simulations to obtain the time history of the modal contributions to the heat current operator. Applying the GKMA method to crystalline silicon (c-Si) and amorphous silicon (a-Si) agrees well with other modal analysis methods and experimental data, which has confirmed its viability and accuracy in describing the mode level details of phonon transport in both crystalline and glassy materials. However, it is acknowledged that other means of decomposing the TC into its modal contributions might also exist.

Here, we briefly summarize the GKMA formalism so that its key features can be emphasized. First, the normal mode eigenvectors are computed from a LD calculation of the entire atomic supercell of the material in question (e.g., at the gamma point–no wave vector). Then, by projecting the atomic velocities from a MD simulation of the same supercell onto the normal mode eigenvectors, one can obtain the time history of each normal mode’s amplitude. Each atom’s instantaneous velocity can then be decomposed into individual mode contributions based on the respective instantaneous normal mode amplitudes, whereby summing the modal contributions returns each atom’s velocity. One then substitutes the modal components of each atom’s velocity into the heat flux operator^23^ to obtain each mode’s instantaneous contribution to the heat flux. The total heat flux can then be obtained from the sum of all individual mode contributions to the heat flux, via


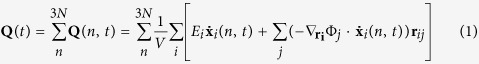


where *n* is mode index, *N* is total number of atoms in the super cell, *V* is volume of the super cell, *E*_*i*_ is the kinetic and potential energy of atom *i*, Φ_*j*_ denotes potential energy of atom *j*, 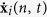 is the contribution mode *n* makes to the velocity of atom *i* and **r**_*ij*_ is distance between atom *i* and *j*. Since the heat flux operator can be used with multi-body potentials^2^,^28^, GKMA could also be applied with multi-body potentials, and as an example we have applied GKMA with ReaxFF^29^ on single polymer chains. Now having access to the individual mode heat fluxes, we can substitute the summation over modes in Eq. (1) directly into the Green-Kubo expression for TC, which describes the TC as proportional to the heat flux autocorrelation function. One then obtains the TC as a direct summation over individual mode contributions,


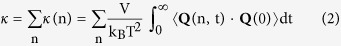


where *κ*(n) stands for TC contribution of mode *n*, k_B_ is Boltzmann constant, *T* is the temperature and *V* is volume. One can substitute the mode heat flux into the second instance of the heat flux autocorrelation function to obtain a double summation over mode-mode cross-correlations, which provides immense insight into the interrelationships and interactions between modes as will be shown later. Using Eq. (2) one can calculate the TC of individual modes in any material where the atoms vibrate around stable equilibrium sites, using one unified formalism. Classical MD has been considered to be inaccurate at low temperatures (i.e., below a material’s Debye temperature) because it does not reproduce the proper mode amplitudes that correspond to the quantum occupations. As a result, classical MD results in a constant heat capacity with respect to temperature, since every mode is equally excited at all temperatures. However, once each individual mode’s TC is obtained, one can easily apply a quantum specific heat correction, which allows one to extend the MD based predictions to essentially any temperature. To obtain the more accurate temperature dependence, one can then use the following expression,


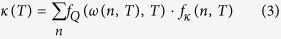


which includes three explicit functions of temperature, namely *f*_*Q*_, *f*_*K*_ and *ω*. In Eq. (3) the function *f*_*Q*_ represents the ratio of the quantum to classical specific heat for mode *n*, which has frequency *ω* at temperature *T* and is unitless. The function *f*_*K*_ represents the GKMA derived mode TC contributions (e.g., it has the units of TC), obtained from MD simulations at discrete values of *T*, where the MD simulations are performed. The function *ω* represents the phonon frequency of mode *n*, which itself also exhibits some temperature dependence. Later, we show how TC prediction improves as each aspect of the temperature dependence is included, which will henceforth be referred to using the subscripts *Q*, *κ*, and *ω*. It should also be noted that thus far in testing the GKMA method, we have only applied Eq. (3) to amorphous materials thus far and have yet to apply it to a crystalline material.

The first and most important source of temperature dependence is in the quantum to classical specific heat ratio *f*_*Q*_, which is what causes the TC to decrease to zero as *T*→0 K for amorphous materials. Furthermore, it restricts the contributions of the high frequency modes at low temperatures and modulates the MD derived TC contributions determined from the GKMA method. The second important source of temperature dependence enters through the GKMA derived TC contributions *f*_*K*_. As temperature changes, the modal interactions change and the contributions of different modes are inherently temperature dependent via the anharmonic nature of the interactions. However, unlike the quantum specific heat correction, which is a continuous function of temperature, MD simulations are run at discrete temperatures. To then generate a piece-wise continuous function for TC vs. temperature, one can interpolate the data for *f*_*K*_ at discrete values of temperature. Here, one can use the data at a few initial temperatures and determine by inspection, what temperature ranges may require additional simulations to improve the resolution of the temperature dependence, in temperature ranges where the contributions change more rapidly. This is because it is advantageous to minimize the number of temperatures needed for *f*_*K*_ to minimize computational expense. Lastly, the phonon frequencies (*ω*) can slightly change with temperature, due to anharmonicity (e.g., frequency softening) and thermal expansion^19^. All of the simulations are performed using constant volume, hence thermal expansion does not play a role, but anharmonic effects can still cause the mode frequencies to change. The extent of the frequency shift as a function of temperature can be easily determined by interpolation of the data at discrete temperatures, using the peak frequency obtained from a Fourier transform of the mode amplitudes.

Using the GKMA method, we then calculated the modes and their respective contributions to TC for a-SiO_2_. All details associated with the calculations are given in the Supplementary Materials. The inverse participation ratio (IPR) and phonon density of states (DOS) are shown in Fig. 1(a,b). The IPR quantifies the extent of localization for a given mode^25^. From Fig. 1(b), there are two regions that have localized modes, from 25 to 30 THz and above 35 THz. Given their higher IPR, we classified both of these groups of modes as locons^25^, which are spatially localized and typically only involve a small group of atoms in the vibration (examples of locon normal mode shapes are given in Supplementary Information). Interestingly, the modes between 30–35 THz are diffusons, which are delocalized over the entire system. Thus, a-SiO_2_ appears to exhibit two regions of locons^27^, which is distinctly different from a-Si^25^. The color-shaded regions in Fig. 1(b) represent the quantum specific heat suppression function multiplied by the DOS, 
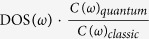
 at three temperatures (100 K, 400 K, and 800 K). Since the classical volumetric specific heat is constant, the area under the black DOS curve is proportional to the specific heat in the Dulong-Petit limit. The areas of color-shaded zones denote the quantum specific heat at the three temperatures. As the temperature increases, there are continuously more modes excited above their ground states, such that they can contribute to the specific heat. Once this happens, these modes start to contribute to TC and as a result, the TC of a-SiO_2_ is continuously increasing from 10 K to 1200 K. Figure 1(c) shows the TC accumulation with frequency at different temperatures. The accumulations above 400 K are very similar, which indicates that the anharmonic effects do not drastically change the mode-mode interactions above 400 K. The increase in TC above 400 K is mainly due to more diffusons and locons starting to become excited above their ground state after which they are able to contribute to the TC.

Figure 1(c,d) shows the TC accumulation vs. frequency before and after quantum specific heat corrections at five different temperatures for a-SiO_2_. Figure 2 shows how the predictions improve, as more accurate temperature dependent information is included. Initially the TC is calculated directly from classical MD, and as expected, the temperature dependence qualitatively differs from the experimental data^30^ as shown in Fig. 2(a). The data in Fig. 2(a) corresponds to evaluating Eq. (3) and setting *f*_*Q*_ = 1. for all modes and it should be noted that we averaged over 10 ensembles for each data point in Fig. 2(a) for the GK calculation. However, after the quantum specific heat correction is applied to GKMA results, the overall experimental trend is obtained, but the results still differ significantly if only the GKMA accumulation is used for a single temperature. Figure 2(b) corresponds to evaluating Eq. (3) with *f*_*Q*_ equal to the quantum to classical specific heat ratio, but the temperature dependence of both *f*_*K*_ and *ω* is neglected, as we have only used the values of *f*_*K*_ at 400 K from GKMA and harmonic frequencies *ω*_0_ at 0 K from LD. In reality, the accumulation, which is obtained from the TC contributions *f*_*K*_, itself is a function of temperature, as indicated by Fig. 1(c). The accumulation, however, only exhibits moderate temperature dependence, which can be roughly approximated by linear interpolation at a few key temperatures. Thus, once the quantum correction (*Q*) and the accumulation (*κ*) temperature dependence are applied, the agreement with experiments improves significantly. We then correct for the temperature dependence of the phonon frequencies (*ω*) themselves (e.g., softening), which can be determined from a Fourier transform of each mode’s kinetic energy^19^. This softening tends to shift the frequencies lower by ~10% at 800 K and 6% at 400 K, which is important, because the quantum correction is sensitive to the mode frequencies. In Fig. 2(c), the TC predictions shift slightly to the left, once the frequency softening *ω*(*n*, *T*) is incorporated into the calculations. In Fig. 2, we have used the subscripts *Q*, *κ*, and *ω* to denote the quantum specific heat temperature dependence, TC accumulation temperature dependence and mode frequency dependence respectively. GKMA^23^ fully includes anharmonicity resulting in different quantitative predictions than the A-F method, which computes the mode diffusivity based on harmonic approximation [25] and assumes the mode diffusivity is not temperature dependent. Instead of using temperature independent thermal diffusivity, we have interpolated the values at several temperatures to incorporate the temperature dependence of *f*_*K*_.

After all the temperature dependence and interpolations have been accounted for, the magnitude and trend of the temperature dependent TC exhibits excellent agreement with experiments as shown in Fig. 2(d). We thus recommend that all three temperature dependencies should be included to obtain the most accurate predictions using Eq. 3. In Fig. 2, we have included the uncertainty associated with sampling a limited number of ensembles, which is given as the standard deviation of the GK results at that temperature. We acknowledge that for a crystalline material, the coupling between modes is dependent on their respective amplitudes. For example, if one simply imagines the time required for a mode to couple to others. Intuitively, the time scale would be very long if no other modes are excited, but if many other modes are highly excited, the coupling/relaxation time is much shorter. Turney and McGaughey^31^ have shown this systematically, and the effect is also quite intuitive. However, we argue that there are instances where this effect is negligible and as a result, only the quantum correction on specific heat is needed to reproduce experiments properly. Thus one cannot expect to apply intuition and expectations based on crystals to amorphous materials, since the behavior is fundamentally different. Hence the ensuing argument is that the quantum correction on specific heat alone works well for amorphous materials, because the quantum effect on “scattering rates” is negligible for such materials. Furthermore, by applying the quantum correction to the 2D mapping of mode-mode correlations (see Eq. 1 in Supplementary Materials), we eliminate the effects of high frequency and low frequency mode interactions, as they would manifest in the heat flux correlation contributions. This in essence eliminates the effect that high frequency modes have on low frequency modes, which is incorrectly included in a classical MD simulation. Comparing with other methods, GKMA demonstrates much better agreement with experiments for a-SiO_2_, as shown in Fig. 3, which derives from the more complete inclusion of the mode dependence, anharmonicity and its temperature dependence.

One of the most striking features of Fig. 1 is the contribution associated with locons. Previously, locons have been thought to exhibit negligible contributions to TC^32^,^33^, due to their restricted spatial extent, which renders it difficult to imagine how they can transfer a significant amount of heat to another location. Despite the long held belief that locons are negligible, Fig. 3 shows that they contribute significantly to the TC of a-SiO_2_. It is important to acknowledge, however, that Alexander and coworkers^34^,^35^ proposed a different theory for non-propagating mode contributions based on fracton hopping. Their theory describes the contributions from fractons, which conceptually must include both diffusons and possibly locons, in terms of their lifetime (e.g., scattering). However, their work only examined the contributions at low temperatures 10–100 K, where the locons are not even excited. Hence the fractons they studied were really only diffusons in most cases. Furthermore, their perspective is fundamentally different than the GKMA viewpoint, which casts their contributions in terms of correlation, as can be seen from each respective derivation^23^,^34^,^35^. The phonon fracton model results in a generalized contribution from fractons that is linear with temperature and has mostly been applied at cryogenic temperatures^35^, where the locon contribution is effectively zero, due to their suppressed heat capacity. This result is then general and would suggest that fractons contribute strongly in all amorphous materials. The GKMA formalism, on the other hand, naturally includes the mode character and anharmonic interactions, which are specific to a given material. As a result, GKMA shows excellent agreement with experiments on both a-Si and a-SiO_2_, but the locon contributions in a-Si are negligible, while in a-SiO_2_ they are not.

Most notably, even after quantum correction, locons are responsible for approximately half of the continual rise in TC for a-SiO_2_ above room temperature. In Fig. 3, the TC contributions vs. temperature with and without the locon contributions included are shown. Without the contributions from locons, the TC begins to deviate from the experiments after 250 K. Furthermore, the cross-correlation maps at different temperatures (see Figs s1 and s3 in the Supplementary Materials) show that even though the autocorrelation appears dominant at all temperatures, the cross-correlation contribution increases with temperature and comprises the dominant portion of the locon contributions. This behavior suggests that modes interact more strongly with other modes of differing frequency as temperature increases, which is consistent with our intuitive understanding of anharmonicity.

The locon contributions come from both cross-correlations and auto-correlations, but it is not entirely clear how these modes are able to help transfer energy from one location to another. One interpretation, motivated by the fact that the cross-correlation contributions to TC for locons, as shown in the Figs s3 and s4 in the Supplementary Materials, is that locons serve as bridges between other modes in regions of the material where atoms are over coordinated and therefore require localized solutions to the equations of motion. As shown in Fig. 4, the correlation between propagons and locons are negligible. However, the correlations between diffusons and locons, and between locons and locons, comprise 55% and 45% of the locon thermal conductivity respectively. From this perspective, even though locons may not move the energy over a significant distance by themselves, they may serve as critical bridges between modes that do, suggesting that their contributions to TC rely on collaboration with other modes. When these modes are excited at high temperature, they help to bridge the energy transfer between diffusons that have vibrations on surrounding atoms. Given that localization causes the motions of some atoms to be largely described by a smaller subset of modes, we then calculated the normalized harmonic energy^36^ associated with locons on each atom in the system. As shown in the Fig. s5, more than 2% of the atoms have more than 40% of the total harmonic energy carried by locons. These locons must somehow couple to other modes to participate in the heat conduction, as Fig. s4 shows that the predominant contributions from locons arise from the locons with the larger participation ratios.

A previous GKMA study of a-Si^23^ showed that locons have negligible contribution to the TC, consistent with previous intuition and A-F also reached a similar conclusion for a-Si using the harmonic approximation^26^. Based on their work, researchers have generally neglected the locon contributions to the TC of amorphous materials^25^,^27^,^32^. However, using GKMA for a-SiO_2_, we find that locon contributions are non-negligible. We believe this is primarily due to the fact that locons constitute 18% of the modes in a-SiO_2_, which is 6X greater than a-Si (3%). If such a large portion of the spatially localized modes in a material does not interact with other modes, then nanometer scale inhomogeneity in the temperature field during steady state heat conduction could arise, due to the inability to conduct heat in certain locations. Such a phenomenon would be rather unphysical and thus it is much more plausible that locons simply exchange energy with surrounding modes and do in fact substantially participate in heat conduction, once excited.

Here we applied the recently developed GKMA method to a-SiO_2_, and have incorporated three sources of temperature dependence. Our results indicate that in order to obtain the accurate predictions, one should incorporate a quantum correction on the heat capacity, the temperature dependence of the GKMA thermal conductivity contributions as well as the softening of the mode frequencies themselves. With these effects included, we have demonstrated the best agreement with experiments to date, as compared to all previous models. Further examination of the contributions from different types of modes revealed quantitative evidence that localized modes (e.g., locons) can contribute significantly to the total TC of a material. In a-SiO_2_, locons are responsible for approximately half of the rise in TC above 400 K. Further study is needed to develop a new physical picture that can describe how both locons and extended, but non-propagating modes such as diffusons are able to transfer heat through disordered materials.

## Additional Information

**How to cite this article**: Lv, W. and Henry, A. Non-negligible Contributions to Thermal Conductivity From Localized Modes in Amorphous Silicon Dioxide. *Sci. Rep.* **6**, 35720; doi: 10.1038/srep35720 (2016).

## Supplementary Material

Supplementary Information

## Figures and Tables

**Figure 1:**
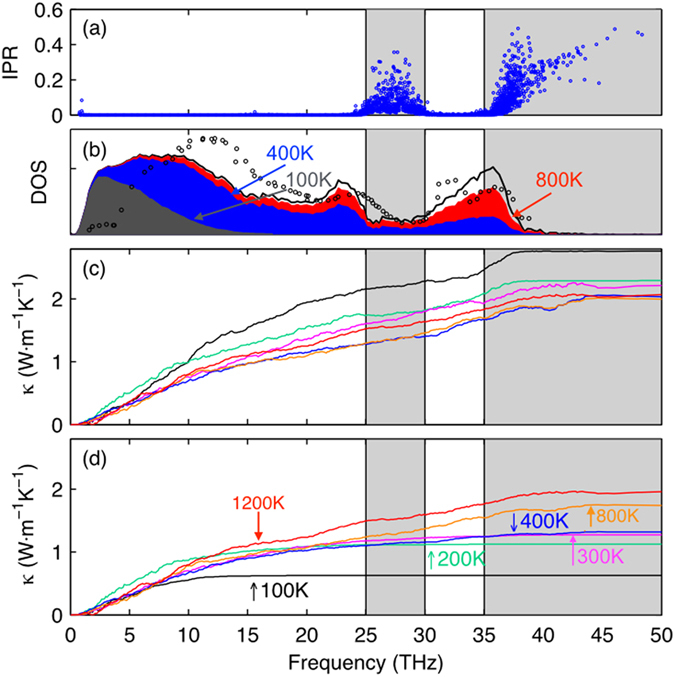
(**a**) IPR of modes in a-SiO_2_; (**b**) Phonon density of states (solid line) and experimental results (black circles)^37^. The three color-shaded areas demonstrate how Bose-Einstein statistics suppress the heat capacity associated with certain modes, which was calculated by multiplying the quantum correction 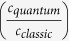 times density of states. The red, blue and gray regions represent 800 K, 400 K, and 100 K respectively and the suppression is significant for 100 K; (**c**) TC accumulation vs. mode frequency for a-SiO_2_ using GKMA at different temperatures (100 K, 200 K, 400 K, 800 K, 1200 K) without the quantum specific heat correction; (**d**) with quantum specific heat correction. The gray shaded areas represent locons.

**Figure 2:**
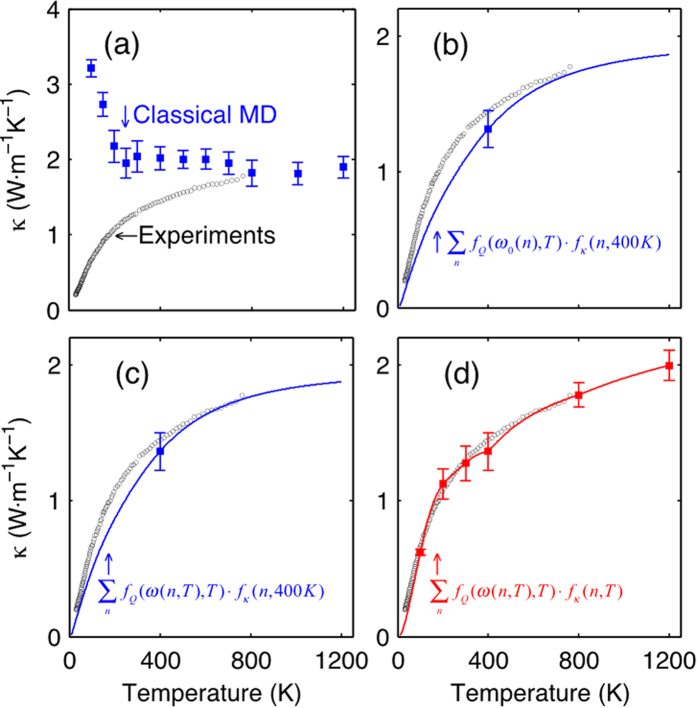
(**a**) shows the GK results with error bars comparing with experiments (black circles); (**b**) is the result of using 400 K GKMA data with quantum specific heat correction; (**c**) is based on the results in (**b**) with the addition of temperature dependent frequencies at 400 K; (**d**) shows the TC using GKMA results at 100 K, 200 K, 300 K, 400 K, 800 K and 1200 K (interpolated in between) with the quantum specific heat correction and temperature dependent frequencies.

**Figure 3:**
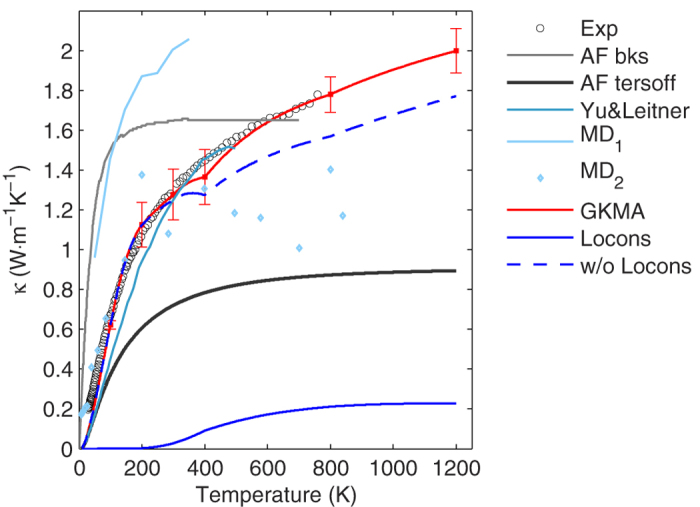
Thermal conductivity vs. temperature as compared to other models and experiments. The red curve is the temperature dependent TC of a-SiO_2_ from GKMA calculations with error bars showing the standard deviation between independent simulations, experimental results (black circles) is from ref. 27, AF bks is Allen-Feldman theory prediction from reference^27^ using Beest-Kramer–van Santen (BKS) potential^38^,^39^, AF tersoff is A-F theory prediction using Tersoff potential, the Yu & Leitner results were obtained from ref. 40, where they used the wave-packet method to calculate the mode diffusivity under the harmonic approximation, MD_1_ is a non-equilibrium molecular dynamics simulation result from Shenogin *et al.*^27^, MD_2_ is the MD result with quantum corrections from Jund and Jullien^41^, the solid blue curve represents the locon contribution to the TC and the dashed blue curve is the sum of all other delocalized mode contributions.

**Figure 4:**
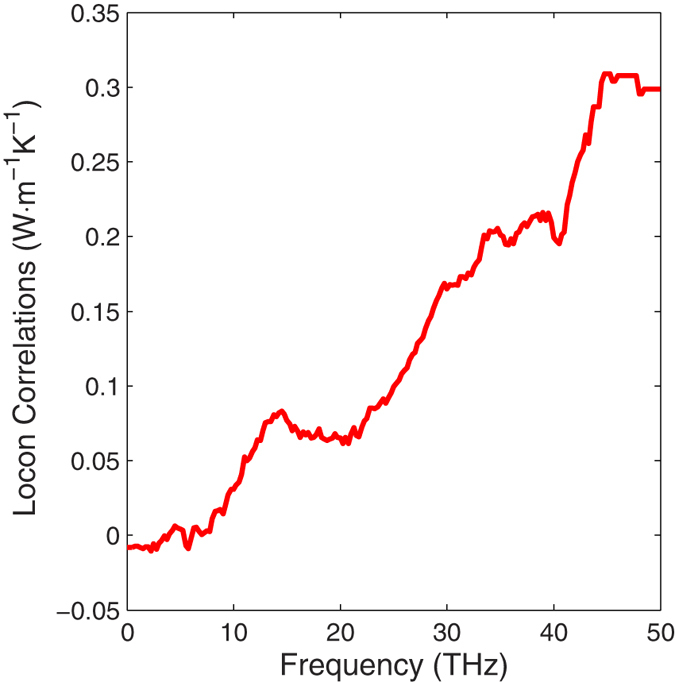
Locon correlations with different frequency modes. The locons-locons correlations contribute around 45% and the diffusons-locons correlations contribute the other 55%. The propagon-locons correlations contribute negligible. Here, each group’s correlation is computed by simply summing the associated values of auto and cross correlations multiplied by the pre-factor in Eq. 2 that yields the units of thermal conductivity. These thermal conductivity contribution values are then shown here, vs. the mode frequency for the modes the locons are correlated with.
