# Supplementary material for: Non-negligible Contributions to Thermal Conductivity From Localized Modes in Amorphous Silicon Dioxide
Source: Sci Rep. 2016 Oct 21;6:35720. doi: 10.1038/srep35720 (PMC5073306; doi:10.1038/srep35720)
Supplement: Supplementary Information [file srep35720-s1.pdf]

Non-negligible Contributions to Thermal Conductivity From Localized Modes in Amorphous Silicon Dioxide

Wei Lv[1], Asegun Henry[*,1,2]

Affiliations:

[1]George W. Woodruff School of Mechanical Engineering, Georgia Institute of Technology, Atlanta, GA 30332, USA.

[2]School of Materials Science and Engineering, Georgia Institute of Technology, Atlanta, GA, 30332, USA.

[*]Corresponding author: ase@gatech.edu

Simulation details

The atomic interactions between amorphous silica atoms were described by Tersoff potential with parameters published by Munetoh et al.[1]. The a-SiO_2 structure was generated by the melting-quenching method. The detailed procedures for generating a-SiO_2 from crystalline silica have been described by Ong *et al.*[2] using the Tersoff potential. After quenching, the structure was annealed at 1100 K for 10 ns to avoid the meta-stability reported by Larkin *et al.*[3]. After generating the a-SiO_2 structure, we calculated the density of states (DOS), and compared with experiments[4]. The agreement is overall reasonable, although there is significant discrepancy at lower frequencies. Nonetheless, Fig. s1 shows that even though there is some discrepancy at low frequencies the specific heat as a function of temperature is still well reproduced.

After we obtained the a-SiO_2 structure, we applied lattice dynamics (LD) at gamma point ($k = 0$) for the supercell (4608 atoms) with periodic boundary conditions to obtain the normal mode eigen values and eigen vectors which allow one to visualize the normal mode shapes. The LD calculations were performed using the General Utility Lattice Program (GULP)[5]. Before LD calculation, one needs the relaxed structure, which is computed at 0K with zero pressure. The supercell is approximately cubic has 4608 atoms, with a length of ~ 40.44 A. The density for the relaxed structure is 2317 Kg/m^3, which is 4% larger than the experimental value 2220 kg/m[36]. The eigenvectors and harmonic frequencies are then obtained using GULP.

Once all the eigen-vectors have been calculated, we read them into the molecular dynamics (MD) simulation in order to calculate the mode level thermal conductivity contributions using GKMA. All MD simulations were performed using the Large Atomic/Molecular Massively Parallel Simulator (LAMMPS) with a time step of 0.1 fs. After equilibrated for 100 ps at 300K using NVT (constant number of atoms, volume, temperature), the heat flux, and mode heat flux were captured for another 2 ns (2 x 10^7 time steps) using NVE (constant number of atoms, constant volume, constant energy). After supplying the eigenvectors once at the beginning of the MD simulation, one is able to obtain the heat flux and kinetic energy of each mode. The integral of the heat flux autocorrelation function is cut off at 30 ps[7] since the largest relaxation time in the supercell is less than 10 ps. After the modal heat flux is computed, the modal thermal conductivity is determined by calculating the correlation between modal heat flux and the total heat flux.

There are a few schemes to reduce the computational cost of GKMA. The first way is to combine a group of modes together. Without calculating individual mode heat fluxes separately, one can combine a group of mode's contributions to the velocity of a given atom together and then substitute it into the heat flux operator. Next, one can sum of a group of n modes' heat flux once instead of as n separate individual contributions. Mathematically, the correlation between combined mode heat fluxes and the total heat flux is exactly equal to the sum of all of the correlations between individual heat fluxes and the total heat flux. The only difference this combination scheme will make is on the specific heat correction. We have to take the averaged frequency of the combined modes in one interval to calculate the specific heat suppression. Here, the frequency interval we used is ~ 0.15 THz, which is small enough to have negligible effect to the final system TC. We have also tested that when using smaller frequency interval, the GKMA results do not change.

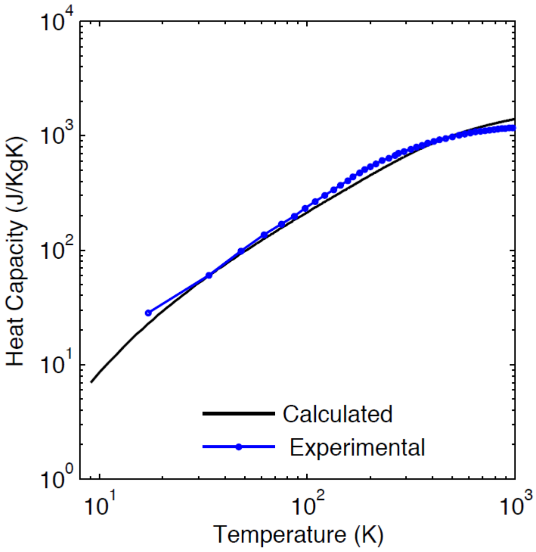

Figure s1. Heat capacity of a-SiO2 from the Tersoff potential, as compared to experimental results [8].

Another scheme is to reduce the frequency of the mode heat flux calculations. Although the simulation time step is 0.1 fs for amorphous silica (a-SiO_2), we do not need to calculate mode heat flux at every time step. When the heat flux is calculated every 5 fs, we observed no difference in thermal conductivity as compared to when a smaller time step was used. In order to efficiently conduct the calculation, we parallelized the algorithm of calculating the heat flux, mode heat flux and mode kinetic energy by implementing the algorithm in the force-routine of the Tersoff potential in LAMMPS[9].

<u>Heat flux pair-correlation map</u>

The mode-mode correlations as shown in Fig. s1 were computed from,

$$\kappa(n,n')|_T = \frac{V}{k_B T^2}\int_0^\infty \left\langle \left(\mathbf{Q}(n,t)\sqrt{\frac{C_Q(\omega(n),T)}{C_c}}\right)\cdot\left(\mathbf{Q}(n',0)\sqrt{\frac{C_Q(\omega(n'),T)}{C_c}}\right)\right\rangle dt \quad (1)$$

where n and n' represent two modes, k_B is Boltzmann constant, T is the temperature and V is volume, $\mathbf{Q}$ is heat flux for a mode, C_Q is quantum specific heat from Bose-

Einstein statistics[10], and C_c is classic specific heat, and ω is frequency of the mode. Using the summation of all correlation functions between pairs of modes $\sum_{n}^{3N}\sum_{n'}^{3N}\kappa(n,n')$, the resulting sum yields the Green-Kubo thermal conductivity at the simulated temperature, which represents the total integrated volume of the 3D map in Fig. s1.

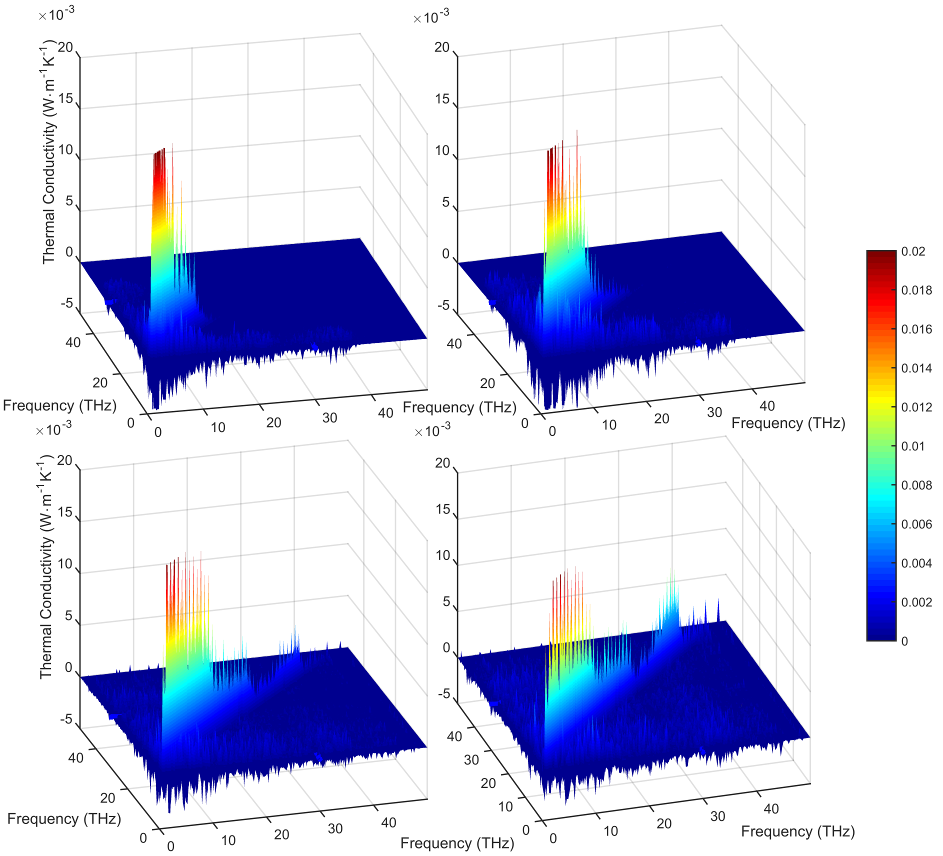

Figure s2. (a) Cross-correlation map of thermal conductivity contributions including the quantum specific heat correction. The values are determined from the mode-mode cross-correlations for a-SiO_2 at 100K, (b) at 200K, (c) at 400K and (d) at 800K.

Total Heat flux Autocorrelation Function

Figure S3 shows total heat flux auto–correlation function and its integral with time separation. The auto-correlations decay very fast. The integral, which is proportional to the thermal conductivity, converges in less than 30 picoseconds.

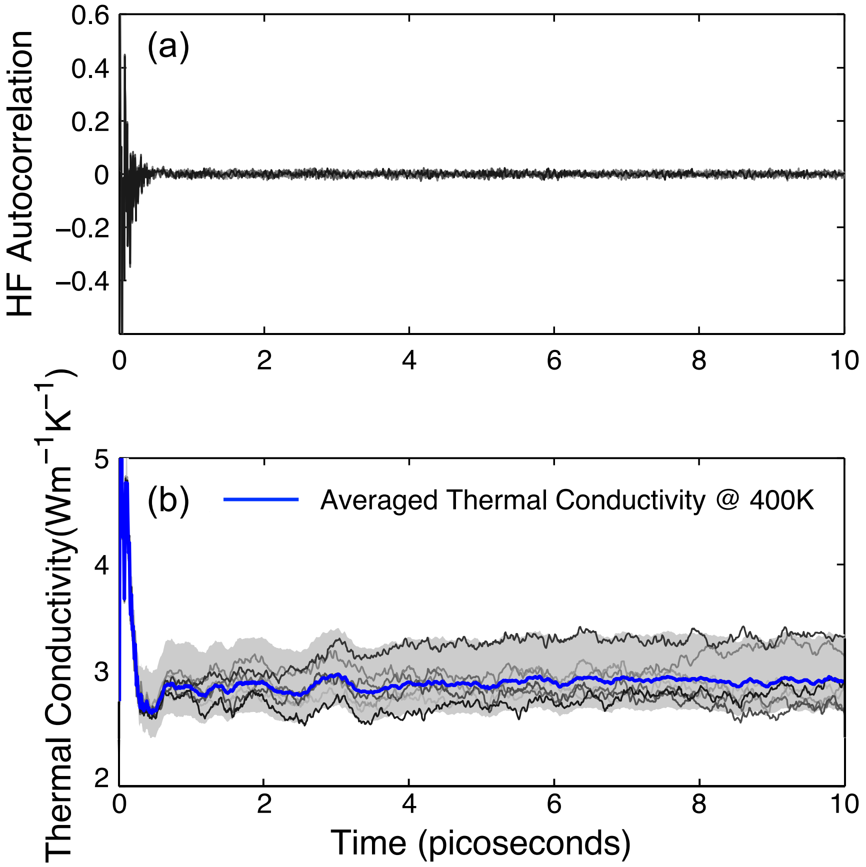

Figure s3. (a) Total heat flux autocorrelation functions (10 ensembles) and (b) integrations with time (in units of thermal conductivity) at 400K. The blue curve in (b) represents the averaged thermal conductivity from ten ensembles. The individual ensemble results are plotted as the grey curves, the shaded region represents the confidence intervals for the ensemble averaging. The width of the interval indicates the degree of certainty (12%).

<u>Locons correlation 3D mapping plot and accumulation plot</u>

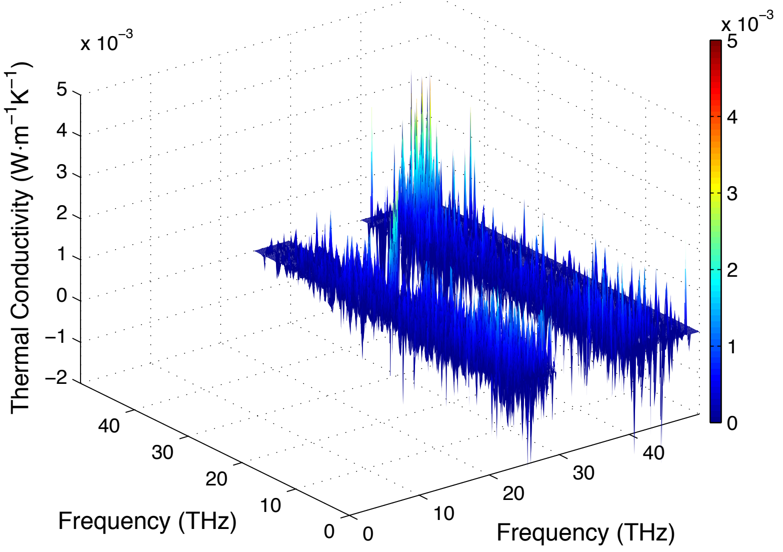

Figure s4. The 3D correlation plot for locons only at 800K.

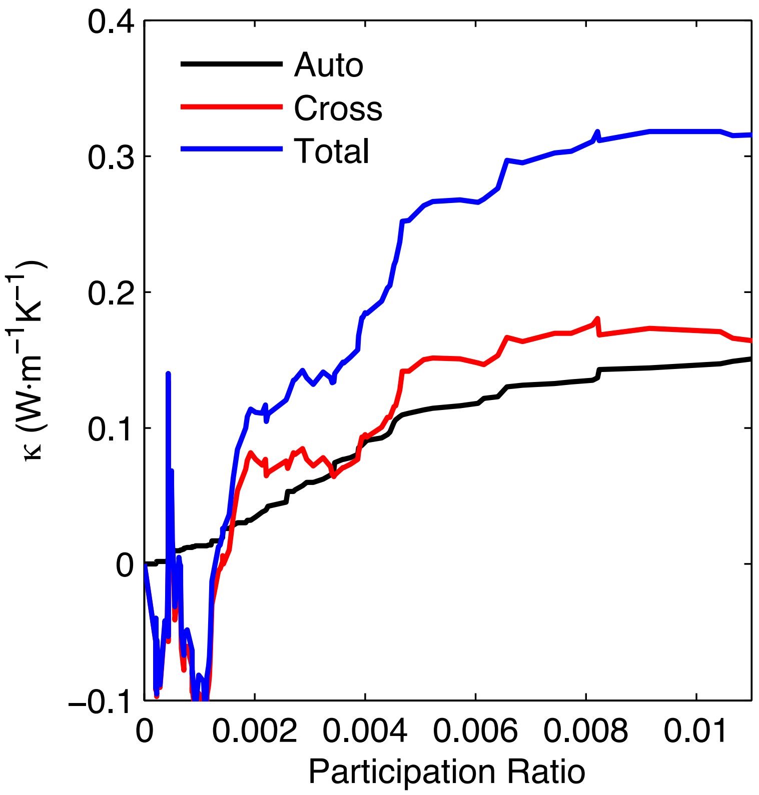

Figure s5. Thermal conductivity contributions from locons that are composed of less than 1% of the system's participation, showing the respective contributions from autocorrelations and cross-correlations.

Locon harmonic energy distribution

The harmonic energy attributed to each atom for each mode is proportional to the magnitude of the eigenvector for each mode on each atom. The detailed derivation and formula are discussed in a recent paper by Gordiz and Henry[11].

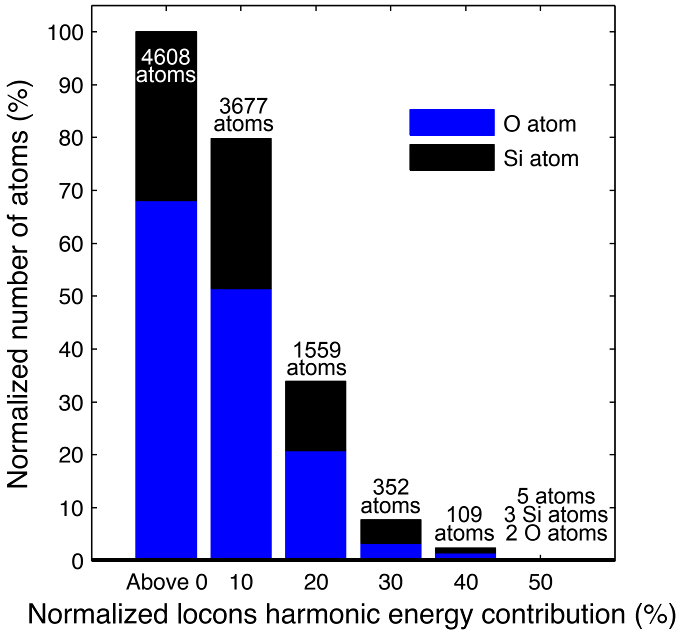

Figure s6. The percentage of number of atoms with different normalized locon harmonic energy contributions. The system has total 4608 atoms; 80% of these atoms

have more than 10% of their energy attributed to locons; 35% of the atoms have more than 20% of their energy attributed to locons; 8% of the atoms have more than 30% of their energy attributed to locons; 2% of the atoms have more than 40% of their energy attributed to locons; and 5 atoms in the system have more than 50% energy attributed to locons.

References

1. Munetoh, S., Motooka, T., Moriguchi, K. & Shintani, A. Interatomic potential for Si--O systems using Tersoff parameterization. *Comput. Mater. Sci.* **39,** 334–339 (2007).
2. Ong, Z.-Y. & Pop, E. Molecular dynamics simulation of thermal boundary conductance between carbon nanotubes and SiO_{2}. *Phys. Rev. B* **81,** 155408 (2010).
3. Larkin, J. M. & McGaughey, A. J. H. Thermal conductivity accumulation in amorphous silica and amorphous silicon. *Phys. Rev. B* **89,** 144303 (2014).
4. Carpenter, J. & Price, D. Correlated Motions in Glasses Studied by Coherent Inelastic Neutron Scattering. *Phys. Rev. Lett.* **54,** 441–443 (1985).
5. Gale, J. D. GULP: A computer program for the symmetry-adapted simulation of solids. *J. Chem. Soc. Faraday Trans.* **93,** 629–637 (1997).
6. Kaviany, M. *Principles of Heat Transfer*. (John Wiley & Sons, 2002). at <http://books.google.com/books?hl=en&lr=&id=dKI4k-9jK88C&pgis=1>
7. McGaughey, A. J. H. & Kaviany, M. Thermal conductivity decomposition and analysis using molecular dynamics simulations: Part II. Complex silica structures. *Int. J. Heat Mass Transf.* **47,** 1799–1816 (2004).
8. SMYTH, H. T., SKOGEN, H. S. & HARSELL, W. B. Thermal Capacity of Vitreous Silica. *J. Am. Ceram. Soc.* **36,** 327–328 (1953).
9. Plimpton, S. Fast parallel algorithms for short-range molecular dynamics. *J. Comput. Phys.* **117,** 1–19 (1995).
10. Ziman, J. M. *Electrons and phonons: the theory of transport phenomena in solids*. *Endeavour* **20,** (1960).
11. Gordiz, K. & Henry, A. Phonon Transport at Crystalline Si/Ge Interfaces: The Role of Interfacial Modes of Vibration. *Sci. Rep.* **6,** 23139 (2016).
